# Insight and executive functions in acquired brain injury: empirical evidence and theoretical frameworks

**DOI:** 10.3389/fneur.2026.1884495

**Published:** 2026-08-11

**Authors:** Natasha Sigala, Melis Cobandag, Leigh Leppard, Nick Medford

**Affiliations:** 1 Department of Clinical Neuroscience, Brighton and Sussex Medical School, University of Brighton, University of Sussex, Brighton, United Kingdom; 2 Department of Neuropsychiatry, South London and Maudsley NHS Foundation Trust, London, United Kingdom

**Keywords:** acquired brain injury, adaptive coding, anosognosia, executive functions, insight, metacognition, predictive coding, prefrontal cortex

## Abstract

Impaired self-awareness affects 30–50% of patients with moderate to severe acquired brain injury (ABI) and represents one of the most clinically consequential obstacles to effective rehabilitation. Despite substantial evidence that self-awareness and executive functions share neural substrates in prefrontal cortex, the mechanisms linking these two functions remain incompletely specified. This paper reviews the empirical evidence for the relationship between executive functions and insight in ABI, and evaluates four theoretical frameworks: the Cognitive Awareness Model, the Dynamic Comprehensive Model of Awareness, Mograbi et al.’s predictive coding framework, and Duncan’s adaptive coding model. It then proposes a novel integration of the latter two as a mechanistically grounded account of why executive dysfunction and impaired self-awareness co-occur following prefrontal damage. We argue that adaptive coding describes the representational flexibility of prefrontal neurons in coding self-relevant information, whilst predictive coding provides the computational logic, driven by precision-weighted prediction errors, through which this adaptive selection is updated. We conclude by discussing assessment and rehabilitation implications in ABI.

## 1 Introduction

Impaired self-awareness, the failure to recognise the presence or severity of one’s own deficits, is among the most clinically consequential sequelae of neurological and psychiatric disease. First described systematically by Babinski (1, 2), who coined the term anosognosia to characterise the denial of hemiplegia following right hemisphere stroke, the phenomenon has since been documented across a wide range of conditions. In stroke, patients may be wholly unaware of motor, sensory, or cognitive changes arising from the injury (3). In dementia, progressive loss of insight into cognitive decline accompanies frontal and medial temporal atrophy, correlating with reduced metabolism in orbitofrontal and posterior cingulate regions (4). In schizophrenia, impaired insight into illness and its symptoms is one of the most robust predictors of poor treatment adherence and functional outcome, and is associated with structural and functional abnormalities in prefrontal cortex (5, 6). Despite differences in aetiology, phenomenology, and clinical context, these conditions share a common pattern: disruption to higher-order self-evaluative processes that depend critically on prefrontal systems.

Acquired brain injury (ABI), encompassing traumatic brain injury (TBI) and non-traumatic causes including stroke, hypoxia, and tumours, represents the population in which the relationship between impaired self-awareness and executive dysfunction has been most systematically examined, and the population with the most pressing rehabilitation implications. Impaired self-awareness (ISA) affects 30–50% of patients with moderate to severe TBI (7), with estimates suggesting that between 76 and 97% of patients in the early post-acute phase, typically within the first year post-injury, demonstrate at least some degree of ISA (8). In this paper we use the terms insight and self-awareness interchangeably. Both refer to a specific domain of metacognitive functioning. Metacognition in its broader sense encompasses both the monitoring of ongoing cognitive processes and the online regulation of cognitive behaviour. Self-awareness as used here refers more narrowly to the stored self-model, the representation a person holds of their own abilities and limitations, and to whether that model is revised in response to new information. This maps onto Flavell’s (9) distinction between metacognitive knowledge, stable declarative knowledge about one’s own cognitive characteristics, and metacognitive monitoring, the dynamic online tracking of current processing. Both are relevant to ABI rehabilitation, but the fidelity and flexibility of the self-model itself is the primary focus of this review. We focus specifically on the interplay between executive functions and computational and neurophysiological accounts of the self-model. The substantial literature on emotional, motivational, and social-cognitive contributions to impaired self-awareness—including apathy, e.g., Bivona et al. (10), anosodiaphoria, and Theory of Mind, e.g., Bivona et al. (11)—lies beyond the scope of the present review and is addressed only briefly, in Section 4.5, where directly relevant to the evidence considered here.

The clinical significance of ISA is substantial and well established. Patients with poor insight show reduced engagement in rehabilitation programmes, diminished use of compensatory strategies, impaired goal-setting, lower functional independence in activities of daily living, and worse vocational outcomes (12–14). The burden on families and caregivers is correspondingly greater when patients lack awareness of their deficits, resist necessary support, or engage in potentially hazardous activities such as driving or independent financial management (12). Neuropsychiatric rehabilitation is fundamentally a collaborative process, and ISA represents a direct impediment to the active patient participation on which that collaboration depends.

Executive functions (EF) encompass working memory, attentional control, cognitive flexibility, inhibitory control, and response monitoring, and their impairment is a central feature of ABI, particularly following damage to prefrontal and frontal-subcortical circuits (15). Frontal regions specifically mediate elements of EF, including updating, shifting, and inhibition (16, 17). The anatomical co-localisation of self-awareness and EF has been recognised since Stuss (18) proposed the frontal lobes as the neural substrate for self-awareness, and empirical support for this relationship has accumulated steadily (19, 20). In a sample of 104 TBI patients, Busch et al. identified three components of executive function: a higher-order EF factor encompassing self-generative behaviours and cognitive flexibility; working memory; and memory errors (21), each implicating prefrontal systems. In a systematic review of TBI populations, Dromer and colleagues found that ISA was significantly related to impaired executive functions in 12 of 15 studies examined (22), whilst Bogod et al. demonstrated that a measure of self-awareness, the Self-Awareness of Deficits Interview (SADI), correlated significantly with the full range of EF measures assessed, including working memory, inhibitory control, and cognitive flexibility (23).

However, the precise mechanisms linking EF and self-awareness remain poorly specified. Several theoretical frameworks have been proposed, such as the cognitive architecture of the Cognitive Awareness Model [CAM; Agnew and Morris (24)], the Dynamic Comprehensive Model of Awareness [DCMA; Toglia and Kirk (25)], and computationally grounded accounts drawing on predictive coding theory (26) and the adaptive coding model of prefrontal function (27). Each captures important aspects of the evidence, but none provides a fully integrated account of how prefrontal mechanisms link EF and insight in ABI, nor a clear translational bridge to clinical practice.

In this review we appraise the evidence for the relationship between executive functions and self-awareness in ABI, and propose a synthesis of this empirical evidence with theoretical frameworks for self-awareness and the structure, function, and connectivity of the prefrontal cortex. We give particular attention to Mograbi et al.’s (26) predictive coding framework and to Duncan’s adaptive coding model (27), arguing that these two accounts are complementary: predictive coding specifies the computational logic through which self-relevant information is selected and updated, whilst adaptive coding describes the neural representational mechanism through which this occurs in prefrontal cortex. By integrating these perspectives, we aim to provide a more complete account of the EF-insight relationship in ABI, and to support better assessment and rehabilitation.

## 2 Theoretical frameworks

Four theoretical frameworks have shaped thinking about the relationship between executive functions and self-awareness in ABI. We review each in turn before proposing an integration.

### 2.1 The cognitive awareness model

The Cognitive Awareness Model (CAM), proposed by Agnew and Morris (24), conceptualises self-awareness as dependent on intact executive functioning, particularly the capacity for self-monitoring, comparison, and the updating of self-knowledge. According to CAM, effective self-awareness requires four interdependent components: a personal database of knowledge about one’s abilities; incoming information about current performance; a comparator mechanism that detects discrepancies between expected and actual performance; and an updating process through which detected discrepancies revise the personal database. This comparator resides within the central executive system and draws on working memory, attentional control, and cognitive flexibility, while the updating process depends on intact memory consolidation.

CAM’s architecture predicts at least two dissociable forms of impaired self-awareness, corresponding to failure at different stages of this sequence. *Executive anosognosia* arises when the comparator mechanism itself is disrupted: mismatches between the personal database and current performance are undetected, typically following damage to the prefrontal executive system. *Mnemonic anosognosia* arises further downstream, when a mismatch may be detected online but fails to be consolidated into the personal database: the patient can, in the moment, register that something is wrong, but this does not lead to revision of their standing self-model. The CAM model has proven clinically influential, providing a rationale for interventions that make performance discrepancies more salient, for example through structured feedback, video review, or prediction-performance comparison tasks (28), calibrated to which stage of the CAM sequence is disrupted.

A neuroanatomically specified extension of the CAM framework has been proposed by Stuss and colleagues (29) and elaborated by Amanzio et al. (30), who situate the CAM‘s executive-monitoring components within a four-level cortico-subcortical hierarchy. In this account, information is processed in a bottom-up sequence from sensory and perceptual levels through basic executive functions to executive-metacognitive functions, with the medial prefrontal cortex (MPFC) and its core hub in the mid-cingulate cortex (MCC) constituting the highest level of conscious self-monitoring. Two prefrontal subsystems are distinguished: the dorsolateral prefrontal cortex (DLPFC), which supports control and sequencing of behaviour and mental set formation, and the MPFC, which subserves metacognitive-executive functions, including drive and motivation. Within this framework, the DLPFC-anterior cingulate cortex (ACC) network is specifically implicated in higher-order cognitive control, including set-shifting and response inhibition, whilst a DLPFC-posterior parietal circuit supports action monitoring and working memory (30). In the CAM account, the comparator mechanism resides within this DLPFC-centred executive system, with detected mismatches between the personal database and current performance transmitted to the MPFC, where they reach awareness. The subsequent updating of the personal database depends on intact interaction between this MPFC-mediated detection and medial temporal memory systems.

When the comparator is disrupted, whether by prefrontal lesion, network disconnection, or reduced MPFC-ACC activity, mismatches between the personal database and current performance fail to reach awareness. This is one candidate route to what Mograbi et al. (31) describe as a “petrified self”: a self-model that is frozen because it is no longer updated by incoming performance information. The concept was developed originally in the context of Alzheimer’s disease, where memory impairment leaves patients evaluating their own performance against an outdated, pre-morbid self-representation, and has subsequently been extended to ABI populations within the transdiagnostic CAM account (30). Importantly, the petrified self is defined by this failure to update rather than by a single specified mechanism. Mograbi et al. (31) do not attribute this to a single mechanism. They identify two candidate routes: comparator dysfunction, or a more direct disruption to memory encoding and consolidation, and treat their relative contribution as an open question, rather than one that is already resolved. The comparator mechanism should therefore be understood as one route to a petrified self, not the model’s defining account of it. The consolidation-based route remains a possibility, and still only partially worked out, particularly in ABI, where the relative contributions of comparator and consolidation failure have not been systematically dissociated.

Amanzio et al. (30) review evidence that this failure-to-update pattern appears across Alzheimer’s disease, frontotemporal dementia, and ABI, and argue that because the neuropsychological picture overlaps across conditions, the CAM framework should apply across all three. However, the evidence base is uneven across conditions. The Alzheimer’s disease evidence draws on eight studies, whereas the frontotemporal dementia and ABI evidence each rest on a single study, a structural imaging study and a clinical case description, respectively. The transdiagnostic claim is much better supported for Alzheimer’s disease than for the other two conditions.

Empirical support for CAM derives from correlations between executive function measures and awareness assessments, particularly those involving online performance monitoring. However, this relationship is inconsistent: Dromer et al. (22) found it significant in 12 of 15 studies reviewed, but not in the remaining three, suggesting that comparator dysfunction, though implicated, is neither reliably present nor absent alongside impaired insight. This can be explained by the two-pathway structure outlined above: comparator dysfunction is neither necessary nor sufficient for impaired self-awareness in general, since the mnemonic route to a petrified self can produce impairment independently of comparator function, and an intact comparator does not by itself guarantee accurate self-assessment if downstream consolidation fails. This suggests that CAM underspecifies the neural and computational mechanisms through which comparison and updating occur.

### 2.2 The dynamic comprehensive model of awareness

The Dynamic Comprehensive Model of Awareness (DCMA) (25) was developed as a non-hierarchical extension of the earlier Pyramidal model of awareness proposed by Crosson et al. (32). Crosson et al.’s (32) pyramidal account arranges awareness into 3 tiers of increasing complexity. Intellectual awareness, the stored knowledge that a deficit exists and an understanding of its implications, forms the base. Emergent awareness, the online recognition of problems as they occur during task performance, sits above it. Anticipatory awareness, the prospective recognition that a problem is likely to arise in a given situation, sits at the top. Each level is explicitly held to presuppose the one below it: intellectual awareness is a prerequisite for emergent awareness, while anticipatory awareness requires both intellectual and emergent awareness as antecedent conditions (32, 33).

Toglia and Kirk’s (25) DCMA restructures this scheme. Rather than three hierarchically ordered levels, the DCMA proposes two dynamically interacting components: *metacognitive awareness*, knowledge of task characteristics and of one’s own capabilities, (broadly corresponding to Crosson’s intellectual awareness); and *online self-monitoring*, the patient’s ability to judge their own capabilities and limitations in relation to the current situation. Online self-monitoring has two components: recognising errors as they occur (corresponding to Crosson’s emergent awareness), and appraising current task demands (corresponding to Crosson’s anticipatory awareness). Critically, the DCMA treats the relationship between these components as dynamic and context-dependent rather than as a fixed hierarchy. A patient’s demonstrated awareness can vary across tasks and situations depending on task familiarity, complexity, environmental demands, and feedback availability. Unlike Crosson’s model, where emergent awareness presupposes intellectual awareness, the DCMA does not hold online self-monitoring to presuppose intact metacognitive awareness. This is the specific sense in which the DCMA is “dynamic”: not merely that awareness fluctuates, but that its components can dissociate from one another rather than unfolding in a fixed sequence.

This distinction follows from the conceptual framework outlined above ((25, 32)). Intellectual awareness depends primarily on declarative knowledge of one’s deficits, so it should rely mainly on intact memory systems. Emergent and anticipatory awareness, by contrast, require intact executive control, particularly response monitoring, cognitive flexibility, and working memory. The DCMA predicts that the relationship between cognitive impairment severity and awareness level follows a systematic pattern: more severe impairments affect all three levels. Moderate impairments, however, may selectively compromise anticipatory and emergent awareness whilst sparing intellectual awareness.

This hierarchical account has been particularly valuable in rehabilitation contexts. Interventions can be calibrated to the patient’s awareness level: those with intact intellectual awareness but impaired emergent awareness may benefit from real-time performance feedback, whilst those with more profound global awareness deficits require more foundational approaches that address the knowledge of deficit itself. The DCMA thus provides a clinical staging framework, though it does not specify the neural or computational mechanisms underlying each awareness level.

### 2.3 Predictive coding and self-awareness

Mograbi et al. (26) propose a neurocognitive framework for self-awareness grounded in predictive coding (PC) theory. Within PC, the brain is conceived as a hierarchical generative model that continuously generates predictions about incoming sensory information and updates those predictions on the basis of prediction errors, mismatches between predicted and actual input (34, 35). Prediction errors can be minimised either by revising the internal model (perceptual inference) or by acting to bring sensory input into alignment with predictions (active inference). The precision of prediction errors determines their weight in updating the model.

Mograbi et al. (26, 31) propose that self-awareness is not a unitary capacity but a collection of processes operating at different levels of cognitive complexity, an emergent property arising from interactions between lower-level implicit processes and higher-level explicit representations. They identify six core self-awareness processes divided into two broad categories: bodily self-awareness processes, including interoception, body ownership and proprioception, and agency (the sense of generating one’s actions); and higher-order representational processes, including metacognition, emotional self-regulation, and autobiographical memory. These processes interact dynamically within an Integrated Self-Awareness Model (ISAM), with bodily processes forming a nested foundation on which higher-order representational capacities depend.

Within the PC framework, self-awareness emerges from the interaction between bottom-up sensory data and top-down predictions about self-relevant states. Disruptions can take three forms: top-down alterations, in which distorted priors or beliefs constrain the interpretation of bottom-up signals; bottom-up disruptions, in which altered incoming signals generate distorted beliefs; and bidirectional disruptions, in which concomitant changes in both directions create self-reinforcing cycles. Applied to ABI, damage to prefrontal systems disrupts either the generation of accurate self-relevant predictions, the detection of self-relevant prediction errors, or the precision weighting that determines whether prediction errors influence model updating.

Research on the conjoint functions of the anterior insular cortex (AIC) and the ACC provides important neuroanatomical specification for the interoceptive component of bodily self-awareness—the foundation on which higher-order self-knowledge depends. This interoceptive component is subsequently incorporated into Mograbi et al.’s (26) ISAM framework. Medford and Critchley (36) argue that the AIC and ACC constitute the input and output arms, respectively, of a functional system for self-awareness: the AIC integrates interoceptive and sensory signals into subjective feeling states, whilst the ACC re-represents these integrated signals as a basis for response selection, conflict monitoring, and autonomic regulation. Within the predictive coding framework, agranular visceromotor cortices, including the ACC and the ventral anterior insula, have been proposed as primary sites for generating interoceptive predictions, with prediction errors computed in the mid- and posterior insula propagated back to modify those predictions (37). The ACC, through its involvement in executive control and attention networks, additionally modulates the precision assigned to those errors and coordinates the appropriate response, whether a revision of the self-model or an action directed at restoring predicted bodily states (36, 37). Disruption to this AIC-ACC system following ABI would therefore be expected to impair not only the interoceptive foundations of self-awareness but also the higher-order self-monitoring processes that depend on accurate interoceptive grounding.

The Mograbi et al. (26, 31) framework represents a substantial advance on earlier cognitive models in several respects. It situates self-awareness within a principled computational framework that specifies the mechanisms of prediction, error detection, and belief updating in formal terms. This computational grounding then generates testable predictions about the direction and level at which disruption occurs in different clinical populations. However, it also carries limitations. First, the empirical evidence for the specific integrative mechanisms proposed in the ISAM remains limited: most studies examine individual self-awareness processes in isolation rather than their interactions. Second, the predictive coding framework does not specify the prefrontal circuits and inter-regional pathways implementing self-relevant prediction and error detection to generate differential predictions about the consequences of localised damage. Third, the neural mechanisms through which precision weighting is achieved, and therefore through which some prediction errors drive belief revision whilst others are attenuated, remain poorly specified at the circuit level. The first of these gaps is where adaptive coding, reviewed next, offers a neurophysiologically grounded account of how prefrontal neurons collectively encode self-relevant task demands through flexible, context-dependent population-level representations, and how damage to specific prefrontal circuits disrupts that organisation.

### 2.4 The adaptive coding model of prefrontal function

The adaptive coding model, proposed by Duncan (27) and supported by electrophysiological and neuroimaging evidence, holds that prefrontal cortex neurons exhibit highly flexible response properties. Rather than coding fixed stimulus features or cognitive functions, these neurons adapt to code whatever information is currently task-relevant, reconfiguring their tuning properties based on task demands, instructions, and context (27, 38). This adaptive coding capacity creates a dense, distributed representation of relevant inputs, actions, and outcomes in a global workspace, a representation that can be flexibly updated as task demands change.

Evidence for adaptive coding comes from multiple sources. Electrophysiological recordings in non-human primates demonstrate that prefrontal cell selectivity to stimulus features is task-dependent: the same neuron may code a visual feature in one task context and an entirely different feature in another (39, 40). Functional MRI experiments in humans reveal that the same prefrontal regions are recruited across diverse cognitive demands, working memory, selective attention, task-switching, response inhibition, consistent with a single underlying adaptive mechanism rather than separate specialised systems (39). More recently, studies have shown that prefrontal representations exhibit low-dimensional structure that transforms systematically over the course of a trial, reflecting specific cognitive operations, and that some of these transformational properties are present even in naive animals prior to training, suggesting intrinsic representational flexibility in prefrontal circuits (41, 42).

Within the adaptive coding framework, working memory, selective attention, and cognitive control are not viewed as separate functions but as manifestations of one underlying process: the flexible, context-dependent coding of task-relevant information. Working memory reflects the sustained representation of task-relevant information in the global workspace; selective attention reflects the filtering of irrelevant information from that workspace; and cognitive control reflects the biasing of processing across other brain systems towards task-relevant dimensions.

Bhandari et al. (43) provide the most detailed human fMRI evidence to date for task-tailored adaptive coding in lateral PFC (lPFC). Across two categorisation tasks with structurally distinct input–output contingencies, lPFC selectively represented task-relevant information whilst suppressing irrelevant stimulus dimensions, and the representational format was organised differently for each task—configured in each case to enhance the separability of the dimensions most critical to that task’s demands. Critically, trial-by-trial variability in neural coding along task-relevant dimensions predicted concurrent behavioural performance, establishing a direct brain-behaviour link. These findings confirm that lateral PFC does not maintain a fixed representational format across cognitive demands but actively reconfigures its population-level coding in a task-specific manner—precisely the flexible, context-dependent representational capacity posited by the adaptive coding model.

Wójcik et al. (44) provide direct evidence, from recordings in macaque PFC, that this task-tailored flexibility is itself learned rather than fixed. As animals learned a new rule from scratch, their prefrontal activity patterns changed across three stages. Early on, the neural representation of the task was broad and unstructured, as though many possible interpretations of the task were being held open simultaneously. Once the rule was learned, this representation became more selectively tuned to the features that mattered. Once it generalised to new stimuli, the representation became more abstract, no longer tied to the specific stimuli used to learn it. In other words, the brain’s coding of a task becomes progressively more efficient and more transferable as learning proceeds, and this progression is itself something the prefrontal cortex must construct through experience, not a capacity that is simply present or absent. This has a direct bearing on self-awareness after ABI: updating one’s self-model in response to a brain injury is, in effect, a learning problem, requiring the capacity to move from a broad, effortful representation of new, disconfirming information about oneself towards a stable, efficiently-coded, generalisable update to the self-model. If adaptive coding capacity is impaired following prefrontal damage, this normal progression from effortful to efficient self-relevant representation may itself be disrupted, offering one account of why some patients appear able to register a deficit in a given instance without this ever consolidating into a lasting revision of how they see themselves.

Applied to self-awareness, the adaptive coding model generates a parsimonious and testable prediction: self-relevant information (i.e., knowledge of one’s own abilities, performance, and state), is coded by prefrontal neurons when self-monitoring is task-relevant. Damage to prefrontal regions disrupts this adaptive coding capacity, impairing the flexible representation of self-relevant information necessary for accurate self-assessment.

### 2.5 Integration: adaptive coding and predictive coding as complementary accounts

The CAM and DCMA describe impaired self-awareness at the level of clinical and cognitive function; predictive coding and adaptive coding describe candidate computational and neurophysiological mechanisms. These are not competing accounts at the same level, and the integration proposed below is not a claim that the latter supersede the former. Rather, consistent with the field’s own recognition of complementary cognitive, neurophysiological, and biopsychosocial explanatory frameworks for impaired self-awareness (33), we treat CAM and DCMA as descriptions of the phenomenon to be explained, and predictive and adaptive coding as candidate mechanistic accounts of it.

Insight into deficit following ABI presents a theoretically distinctive explanatory challenge: it requires an account not only of how self-relevant information is encoded, but of how that information is selected, weighted, and updated in response to discrepant evidence. We propose that these two demands correspond to separable but mutually constraining levels of neural organisation, and that the adaptive coding and predictive coding frameworks are complementary rather than competing accounts, each operating at a distinct level of neural organisation. The account that follows describes the internal logic of this integration. It is a theoretical model of how executive dysfunction and impaired self-awareness could arise from a shared mechanism, and its specific claims have not yet been directly tested in ABI populations. Predictive coding specifies the computational logic through which self-relevant information is selected and updated: the brain generates hierarchical predictions about self-relevant states, with prediction errors weighted by their estimated precision driving the updating of self-models. Intact insight, on this account, requires that prediction errors generated by discrepancies between anticipated and actual performance carry sufficient weight to revise higher-order beliefs about one’s own abilities. Impaired insight following ABI may therefore reflect a failure at this level: ascending error signals are attenuated or misdirected, such that the self-model is shielded from disconfirming evidence rather than updated by it. Adaptive coding specifies the neural representational mechanism proposed to underlie this process in prefrontal cortex: prefrontal neurons flexibly recode whatever information is currently task-relevant, including self-relevant information when self-monitoring is contextually demanded, such that the population code reorganises around the dimensions most critical for ongoing performance evaluation. On this account, damage to prefrontal circuitry, or to the white matter pathways through which prediction errors reach it, would compromise both the fidelity of self-relevant representations and the capacity of those representations to be revised, which we propose as the mechanism producing the characteristic profile of unawareness that defines anosognosia and related disorders of insight in the ABI population.

Together, the two frameworks suggest that self-awareness emerges from prefrontal systems that both adaptively code self-relevant information and minimise self-relevant prediction errors through hierarchical generative models. A further prediction of this account concerns the precision weighting of self-relevant prediction errors, which determines their influence on adaptive neural coding. This weighting is predicted to be enhanced when self-evaluation is explicitly required, when self-relevant outcomes carry high motivational significance, or when the discrepancy between predicted and actual performance is large, though this has not yet been tested in ABI populations. This integrated account also offers a candidate explanation for dissociations: when damage is partial, context-specific, or compensated by spared prefrontal regions, executive functions and insight can dissociate, reflecting the context-dependent and distributed nature of adaptive coding. However, this explanation for observed dissociations has not been directly tested.

Rather than superseding CAM and DCMA, this integration offers a mechanistic account of the clinical constructs each describes. For CAM, it specifies the comparator function as precision-weighted prediction error detection implemented in prefrontal adaptive coding circuits, and situates the updating process discussed in Section 2.1 as dependent on the interaction between this detection process and medial temporal consolidation. For DCMA, it offers a candidate neural substrate for the two components introduced in Section 2.2: metacognitive awareness and online self-monitoring. Metacognitive awareness maps onto stored self-knowledge in autobiographical and semantic memory systems that interact with prefrontal generative models. Online self-monitoring, encompassing both error recognition and the appraisal of task demands, maps onto online adaptive coding of real-time performance information and associated prediction errors, with the prospective component of task appraisal corresponding to the prospective generation of self-relevant predictions about upcoming performance. The representational geometry framework, e.g., Wójcik et al. (44) and Sigala et al. (45), offers a further mechanistic specification of what could go wrong in ABI, extrapolated from findings in the intact system rather than demonstrated in ABI directly. In the intact system, lateral PFC develops task-tailored geometries in which the most critical output dimensions are encoded abstractly and with enhanced separability, affording robust and generalisable readout. We propose that prefrontal damage in ABI would disrupt these task-tailored geometries selectively, reducing the separability of self-relevant coding dimensions, impairing the suppression of irrelevant information within contextual subspaces, and degrading the orthogonal organisation that protects self-evaluative representations from interference by competing task demands. On this account, this disruption would manifest as the clinical pattern of executive function and insight impairments observed after ABI: a failure not merely of information availability but of the representational precision and structure needed for reliable self-monitoring. This extension of the representational geometry framework to ABI has not yet been directly tested.

## 3 Neuroanatomical foundations

### 3.1 Prefrontal contributions

Converging evidence from lesion studies, structural neuroimaging, and functional neuroimaging implicates prefrontal cortex in both executive functions and self-awareness, with different prefrontal subregions contributing differentially to each. The dorsolateral prefrontal cortex (DLPFC) has been consistently associated with working memory, cognitive flexibility, and response monitoring, executive capacities frequently impaired following ABI (15). Damage to DLPFC correlates with impaired awareness of cognitive deficits across TBI, stroke, and neurodegenerative populations (19, 20). The ventromedial prefrontal cortex (vmPFC) and orbitofrontal cortex are particularly involved in self-referential processing, the integration of interoceptive signals with cognitive appraisals, and the updating of value-based self-evaluations. The anterior cingulate cortex (ACC), particularly its dorsal subdivision, has been implicated in conflict monitoring, error detection, and performance adjustment, the neural operations most directly relevant to online, emergent self-awareness (19).

These regional associations are consistent with the adaptive coding framework’s account of distributed, context-dependent prefrontal function. Ham et al. (19) demonstrated that impaired self-awareness after TBI is associated with abnormal activity in the right dorsal prefrontal cortex. More accurate self-insight, in turn, correlates with increased activation in right anterior dorsal prefrontal cortex during self-evaluation. In a systematic review of neural correlates of ISA after ABI, Terneusen et al. (20) found the anterior cingulate cortex and anterior insula to be the most consistently implicated regions. Disruption to functional interactions within prefrontal networks accounted for ISA more reliably than focal lesion location.

### 3.2 Network perspectives

Systems neuroscience has shifted from modular localisation accounts towards network-level models of large-scale cortical organisation. This shift is especially pertinent for understanding ISA, where the absence of a consistent lesion-deficit correspondence has long resisted region-specific interpretation. Three large-scale networks are relevant here. The frontoparietal control network (FPCN), incorporating DLPFC and intraparietal sulcus, supports adaptive control and task-switching (39). The default mode network (DMN), encompassing medial prefrontal cortex, posterior cingulate cortex, and angular gyrus, is associated with self-referential processing and autobiographical memory (26). The salience network, incorporating dorsal ACC and anterior insula, is involved in detecting behaviourally relevant signals and in switching processing resources between the FPCN and DMN (46).

The role of the salience network in ISA is further specified by evidence for its connectivity structure. Resting state studies reveal two distinct AIC-cingulate subsystems, with the anterior insula preferentially coupled to the anterior and mid-cingulate regions, the same regions most consistently implicated in error detection, conflict monitoring, and performance adjustment (36). Granger causality analyses confirm directed causal influence from right fronto-insular cortex to ACC, establishing a functional hierarchy within the network rather than co-activation (47). This connectivity architecture has specific implications for ABI. Lesions or white matter disruption affecting fronto-insular pathways do not merely reduce salience detection, but selectively degrade the directed signal through which interoceptive and performance-relevant inputs reach the cingulate, which coordinates self-evaluative responses. The consequence is a network-level failure of continuous self-monitoring that reflects disruption to the network’s directed architecture rather than to any single region.

In the context of ABI, Dromer et al. (22) concluded that ISA is not directly determined by lesion location but rather by functional interactions within brain networks, with key roles for the anterior cingulate cortex and anterior insula. Ham et al. (19) showed that poor behavioural adjustment following TBI is associated with reduced functional connectivity between the ACC and inferior frontal gyri within the FPCN. These network-level findings are consistent with two of the frameworks reviewed above. The adaptive coding framework emphasises that self-relevant information is represented in distributed prefrontal circuits rather than dedicated self-awareness regions. The predictive coding framework requires coordinated hierarchical communication across cortical levels for effective prediction and error detection.

### 3.3 Structural correlates

Structural neuroimaging studies in ABI populations provide converging evidence for the role of frontal grey matter in EF. Killgore et al. (48) demonstrated that ventromedial prefrontal grey matter volume correlates with neurocognitive performance following mild TBI. Livny et al. (49) similarly showed that post-injury cognitive deficits across multiple domains are associated with grey matter volume reductions in TBI patients. Converging evidence from normative ageing research indicates that bilateral frontal grey matter volume specifically mediates the common EF component, the general executive capacity shared across updating, shifting, and inhibition. Parietal grey matter, despite also showing age-related decline, does not mediate EF differences (16). This dissociation suggests that frontal grey matter integrity is specifically critical for the general adaptive capacity that underpins executive functioning across domains. Given that the same frontal regions are implicated in self-awareness, structural frontal changes, whether from normal ageing, TBI, stroke, or other ABI aetiologies, may simultaneously compromise both EF and insight through a common mechanism of reduced adaptive coding capacity. Together with the evidence from the review by Terneusen et al. (20) there is some evidence that ISA after ABI implicates a network that partially overlaps with distributed prefrontal systems that support executive functioning, rather than a separable self-awareness module.

## 4 Empirical evidence: executive functions and insight in ABI

### 4.1 Prevalence and clinical impact of impaired self-awareness

Dromer et al.’s (7, 22) two-part systematic review of ISA after TBI provides the most comprehensive synthesis of the current evidence base. Across 95 included studies, ISA was found to occur in 30–50% of patients with moderate to severe TBI, with the prevalence varying considerably depending on assessment method, time post-injury, and the domain of functioning assessed. Longitudinal data indicate that ISA tends to improve over time, particularly in the first year post-injury, though a substantial proportion of patients show persistent impairment (7). Recovery in awareness of behavioural and cognitive domains generally proceeds more rapidly than recovery of awareness of emotional and social changes.

The functional consequences of ISA are pervasive. Dromer et al. (22) identified significant associations between ISA and decreased treatment adherence, reduced rehabilitation effectiveness, impaired goal-setting and attainment, lower functional independence in activities of daily living, poorer vocational outcomes, and increased burden on carers. Eight of ten studies examining the relationship between ISA and injury severity found significant associations, with higher injury severity linked to greater ISA (22).

### 4.2 Heterogeneity in the assessment of self-awareness

A methodological caveat is necessary before reviewing the empirical evidence in detail. Self-awareness has been operationalised inconsistently across the studies synthesised below, using measures that Dromer et al. (7) group into four broad categories: self-proxy rating discrepancy, performance-based discrepancy, structured interviews, and clinician ratings. These categories show only moderate intercorrelation, indicating that they capture partially distinct aspects of self-awareness rather than a single unitary construct. This heterogeneity bears directly on how the EF-insight correlations reviewed in Section 4.3 should be interpreted, since the strength and even the direction of an association can depend on which measurement category a given study employed. Section 5.2 returns to this issue in detail, including the specific evidence for why structured interview and online error-awareness measures show more consistent relationships with EF than discrepancy-based scores.

### 4.3 Executive function domains and their relationship to insight

Across the reviewed literature, response monitoring and error detection show the most consistent and robust relationship with self-awareness. Whilst individuals with impaired error awareness on cognitive tasks frequently demonstrate reduced awareness of functional limitations in daily life (50), this relationship is not uniform: online task-based error awareness and offline awareness of everyday functioning appear to reflect distinct levels of self-awareness that do not always converge (20). This finding aligns with predictions from all four theoretical frameworks. CAM identifies error detection as a core comparator function. The DCMA identifies error recognition, one facet of online self-monitoring, as central to it. Predictive coding identifies errors as the prediction errors that drive self-model updating. Adaptive coding identifies error-related information as task-relevant content coded by prefrontal neurons. The convergence of these predictions with the empirical evidence positions response monitoring as the strongest single link between EF and insight in ABI. Amanzio et al. (30) in their review spanning ABI, Alzheimer’s disease, and frontotemporal dementia confirm that response inhibition, cognitive set-shifting, and action-monitoring are the EF components most consistently associated with reduced self-awareness, lending support to the centrality of these processes and to the generalisability of the EF-insight relationship beyond TBI populations specifically.

Cognitive flexibility, the capacity to shift between mental sets, update task representations, and adapt to changing contingencies, demonstrates robust correlations with self-awareness, particularly with functional awareness relating to instrumental activities of daily living. Deficits in cognitive flexibility have been proposed as a mechanism through which self-awareness is compromised, alongside response inhibition and performance monitoring, by disrupting the executive processes that support accurate self-appraisal (51). Consistent with this, individuals with ABI show particular difficulty updating outdated beliefs about their abilities when confronted with disconfirming evidence: even when able to self-regulate accurately in the moment, they tend to revert to prior beliefs about their performance rather than revising their self-model in light of accumulated contrary experience (52). From the adaptive coding perspective, cognitive flexibility reflects the capacity to reconfigure prefrontal neural representations when task demands change; reduced flexibility thus implies a reduced capacity to update self-relevant representations in response to prediction errors. Dromer et al. (22) found executive functions, particularly inhibition and working memory, to be the cognitive domains most consistently related to ISA across the 12 studies in their review that identified a significant association.

Working memory and sustained attention show moderate correlations with self-awareness across studies, though the relationships are less consistent than for response monitoring and cognitive flexibility. Working memory capacity constrains the simultaneous maintenance of task demands, performance standards, actual performance, and the comparison between them—limiting the resources available for self-monitoring. However, some studies find no significant relationship between working memory capacity and awareness measures, suggesting that capacity limitations alone do not fully account for awareness deficits (53). This pattern is consistent with the adaptive coding framework’s account: working memory is not a separable system but a component of adaptive prefrontal coding, and its contribution to self-awareness depends on the extent to which self-monitoring is explicitly required by the current task context.

Inhibitory control shows the most variable relationship with self-awareness across studies (23, 52). This inconsistency may reflect measurement issues: standard inhibitory control tasks may not capture the specific inhibitory demands relevant to self-awareness, such as suppressing responses to negative feedback or inhibiting outdated self-beliefs. Conceptually, inhibitory control within the adaptive coding framework is not a separable capacity but reflects the operation of the global workspace—the mechanism for making selected information broadly available across cognitive systems—which both maintains task-relevant representations and actively suppresses competing information, including outdated self-knowledge that is inconsistent with current performance.

### 4.4 Injury severity, aetiology, time course, and longitudinal change

The relationship between ISA and injury severity is well established: higher severity injuries, reflected in measures such as Glasgow Coma Scale scores and post-traumatic amnesia duration, are associated with greater ISA (22). This relationship is consistent with the adaptive coding account, which predicts that more extensive prefrontal damage reduces adaptive coding capacity more severely, impairing both EF and self-awareness in proportion to the extent of the damage. However, the relationship is not deterministic: some individuals with severe injuries maintain relatively preserved self-awareness, whilst others with milder injuries show significant ISA. These individual differences likely reflect premorbid cognitive reserve, the specific anatomical distribution of damage within prefrontal networks, and the extent to which compensatory recruitment of alternative prefrontal regions is possible.

Longitudinal data from Dromer et al. (7) indicate that awareness tends to improve over time, particularly in the first year post-injury, and that improvements in awareness of behavioural and cognitive domains precede improvements in emotional and social awareness. This temporal pattern is consistent with the prediction that self-awareness depends on the reestablishment of functional adaptive coding capacity in recovering prefrontal circuits, unfolding gradually and domain-specifically as neural recovery and compensatory reorganisation progress. The finding that ISA can improve through therapy participation itself, even without full initial insight, suggests that structured task engagement may promote the recovery of adaptive coding capacity by providing the contextual demands that drive prefrontal neural reconfiguration.

A related source of heterogeneity, distinct from severity and time course, concerns aetiology. Much of the evidence reviewed above, including the prevalence figures cited in Section 4.1, derives from systematic reviews and primary studies conducted specifically in TBI populations (7, 22); ABI as defined in this review also encompasses stroke, hypoxic injury, and tumour, populations in which the relationship between EF and self-awareness has been considerably less systematically characterised. Terneusen et al. (20) is a notable exception in reviewing neural correlates across ABI populations more broadly, but direct comparisons of the EF-insight relationship across aetiologies within a single study remain rare. Generalisation of the prevalence, correlational, and neuroanatomical findings reviewed here from TBI to other ABI aetiologies should therefore be made cautiously, pending further aetiology-specific research.

### 4.5 Social cognition and emotional factors

Beyond the core EF domains, Dromer et al. (22) identified associations between ISA and difficulties in social cognition, including Theory of Mind and emotion recognition, though this finding rested on a small number of included studies and should be interpreted cautiously. An inverse relationship between self-awareness and mood was considerably more consistent: higher self-awareness correlates with increased anxiety and depression across the majority of studies reviewed, whilst ISA may function as a psychological buffer against distressing recognition of deficits (22). This pattern underlines that ISA in ABI is not simply a cognitive deficit but is embedded in a broader context of emotional regulation and social cognition, domains that are relevant to the predictive coding framework’s account of self-awareness as encompassing emotional self-regulation and higher-order representational processes. A full integration of these emotional, motivational, and social-cognitive contributions, e.g., Bivona et al. (10, 11), with the executive and computational account developed in this review remains an important task for future theoretical development (see Section 6.4).

## 5 Assessment considerations

### 5.1 Assessing executive functions in the context of insight

A significant challenge in this literature concerns the ecological validity of standard EF measures. Conventional neuropsychological tests of executive functions are administered in structured, examiner-led contexts that reduce the self-monitoring demands inherent in real-world performance. Performance on tasks such as the Wisconsin Card Sorting Test or Tower of London may show only modest correlations with executive challenges in daily life. This may underestimate the relationship between executive dysfunction and awareness impairments in naturalistic contexts. Morton and Barker (54) found that implicit cognitive functions contributed to awareness independent of explicit executive measures. This indicates that standard EF batteries do not exhaustively capture the cognitive architecture underlying insight. This implicit contribution may reflect processes more relevant to unstructured, real-world performance demands, as opposed to structured explicit testing, though their design does not directly address this distinction. It remains unclear whether ecologically oriented EF measures, such as the Behavioural Assessment of the Dysexecutive Syndrome [BADS; Canali et al., (55)] or naturalistic observation, demonstrate stronger relationships with awareness than conventional laboratory tests. Attempts to establish such relationships have produced inconsistent findings, with some studies reporting limited ecological validity even for measures specifically designed to capture dysexecutive difficulties in everyday contexts (56). The extent to which context-sensitive assessment might reveal stronger EF-awareness associations therefore warrants further investigation.

The adaptive coding framework suggests a specific assessment implication. Measures that require flexible reconfiguration of task representations, rather than performance under fixed, highly structured conditions, should capture adaptive prefrontal capacity most sensitively, and show stronger relationships with self-awareness. This prediction provides a basis for developing more sensitive assessment approaches.

### 5.2 Assessing self-awareness: multi-method approaches

The theoretical models reviewed in Section 2, particularly the CAM and DCMA, argue strongly for multi-method assessment of self-awareness that captures intellectual, emergent, and anticipatory awareness separately. Bogod et al. (23) provide illustrative empirical support for this approach. Self-proxy discrepancy scores, whilst widely used, conflate genuine unawareness with rating biases and differing interpretive frameworks, and show weak relationships with EF measures. Structured interviews such as the SADI, which assess knowledge of deficits and their functional implications directly, show stronger relationships with EF measures and better predict injury severity and rehabilitation outcomes. Online error awareness measures, assessing whether individuals recognise errors immediately after commission, most directly capture the prediction error detection processes that are central to both the CAM and the predictive coding framework. Critically, online error awareness and metacognitive awareness show distinct neuroanatomical correlates. Frontal lobe lesions selectively impair both online error monitoring and autonomic response to recognised errors, and the two awareness types are not significantly correlated with one another, indicating that they are underpinned by dissociable neural systems (57). Online error awareness measures are therefore the measures most strongly associated with frontal lobe functioning. Their dissociation from metacognitive awareness provides a further argument for multi-method assessment rather than reliance on any single measure.

Assessment should span multiple functional domains—cognitive, motor, emotional, social—given the domain-specific nature of awareness deficits (33). A patient may show intact intellectual awareness of motor limitations whilst demonstrating significant impairment in emergent awareness of cognitive or emotional changes. Beyond domain, assessment should also distinguish between what a patient reports and what a patient does. Bivona and Villalobos (33) propose a further distinction within anticipatory awareness between *declarative-anticipatory* awareness, the ability to describe necessary compensatory strategies without external corroboration, versus *real-anticipatory* awareness, confirmed only when an informant verifies that the patient’s actual behaviour is consistent with what they report. This distinction has direct clinical relevance. A patient may declare that they should not resume driving given known cognitive and behavioural deficits, whilst family members report that the same patient persistently requests the car keys. This discrepancy is invisible to assessment tools that rely on declarative report alone. Domain-specific profiles of awareness have direct implications for rehabilitation planning, identifying which areas of functioning, and which level of a patient’s self-report, are amenable to metacognitive intervention and which require more compensatory or environmental approaches.

## 6 Clinical and rehabilitation implications

### 6.1 Awareness as a prerequisite for metacognitive rehabilitation

A well-established finding in the rehabilitation literature is that metacognitive strategy training, teaching patients to predict, monitor, and adjust their performance, is most effective when patients have at least some degree of emergent or anticipatory awareness of their deficits (58). Patients who lack awareness entirely are unable to engage with the self-monitoring demands of metacognitive interventions, and are better served initially by experiential learning approaches that create the conditions for emergent awareness to develop, such as structured task engagement with real-time feedback in supported contexts (28). The DCMA provides a useful staging framework here, identifying the level of awareness from which rehabilitation should begin. The integrated adaptive and predictive coding account reinforces this clinical principle: metacognitive interventions effectively train the prediction-generation and error-detection processes that drive self-model updating, but require sufficient residual adaptive coding capacity for this training to take effect.

### 6.2 Interventions targeting adaptive capacity and prediction error detection

Evidence-based rehabilitation approaches for ISA in ABI typically incorporate education about brain injury and its effects, self-monitoring training with structured feedback, prediction-performance comparison tasks to highlight discrepancies, experiential learning in supported contexts, and metacognitive strategy training emphasising prediction, monitoring, and adjustment. These approaches map coherently onto the integrated framework: education and structured feedback enhance the salience and precision of self-relevant prediction errors; prediction-performance comparison tasks make discrepancies explicit and unambiguous; and experiential learning in varied contexts promotes the reestablishment of adaptive coding capacity through practice-dependent neural reconfiguration.

The adaptive coding framework suggests a specific rehabilitation principle: training should be conducted across varied contexts and task demands, rather than in highly structured, fixed-format conditions, because adaptive coding capacity is built through contextual variety. Encoding the same information under varied conditions strengthens the flexible representational capacity of prefrontal networks rather than producing narrow, context-specific learning (59). This principle aligns with evidence from motor and verbal learning research demonstrating that training conditions which introduce difficulty and slow apparent rates of acquisition tend to produce superior long-term retention and transfer compared to conditions that optimise performance during training itself (60).

The predictive coding account of ISA suggests that the timing and precision of feedback are critical determinants of whether prediction errors successfully drive self-model updating. Feedback delivered during task performance, when the adaptive coding of performance information is active, is more likely to influence the self-model than feedback delivered after a delay, when the neural representation of the performance has decayed. The motivational significance of self-relevant feedback also modulates its precision weighting and therefore its impact on self-model updating: feedback that carries meaningful consequences for the patient’s valued goals and activities is more likely to drive awareness updating than feedback on abstract or clinically selected tasks. Rehabilitation planning should therefore be attentive to individual patients’ goals and values in selecting the contexts in which feedback is provided.

### 6.3 The bidirectional relationship between insight and EF rehabilitation

The relationship between ISA and EF rehabilitation is bidirectional in principle, though the strength of evidence differs substantially between the two directions. Poor insight reliably limits the effectiveness of EF interventions, by reducing patients’ engagement, motivation, and capacity to apply metacognitive strategies. Whether the reverse holds—that targeted EF rehabilitation, particularly interventions that directly train response monitoring, cognitive flexibility, and adaptive task management, produces improved insight as a collateral benefit by restoring the adaptive coding capacity on which self-awareness depends—is a testable prediction of the integrated framework proposed here, not an established finding, and is evaluated against the available evidence below. For patients with relatively preserved EF but domain-specific ISA, awareness-focused interventions remain the appropriate starting point regardless of how this question is resolved.

Evidence on the reverse relationship is mixed and, on direct test, does not support a simple collateral-benefit account. Villalobos et al. (61) found that inhibition and cognitive flexibility at admission predicted self-awareness level at discharge across a cognitive rehabilitation programme. They note, however, that other studies have reported a null EF-self-awareness association, possibly because neither EF nor self-awareness is a unitary construct, such that the relationship may hold for some EF components or self-awareness domains without generalising across all. More directly, Cantor et al. (62), evaluating the STEP (Short-Term Executive Plus) metacognitive strategy programme in 98 individuals with moderate–severe TBI, found significant improvement in executive function but no accompanying improvement in self-awareness.

Current clinical consensus reflects this. The INCOG 2.0 guidelines (63) do not recommend EF training as a route to improving self-awareness. Instead, they give Level A evidence (i.e., supported by at least one meta-analysis, systematic review, or adequately powered randomised controlled trial) to self-awareness-specific interventions—structured feedback, and video feedback in particular, which outperforms verbal feedback alone—as a distinct treatment target requiring its own domain-specific intervention, alongside metacognitive strategy instruction (e.g., Goal Management Training) for executive impairments. This is consistent with Bivona et al.’s (64) recommendation for concurrent rather than sequential assessment and treatment of EF and self-awareness, and with the guidelines’ explicit observation that self-monitoring “does not cut across domains”—a patient may be self-aware of a memory impairment yet unaware of a reasoning difficulty, requiring awareness-specific intervention for each. Careful assessment of both constructs at the outset of rehabilitation, and regular reassessment throughout, is essential for tailoring interventions to the individual patient’s profile.

This picture is consistent with the most recent systematic evidence available. Eliav et al. (65), reviewing interventions for executive function and self-awareness in the early (subacute) phase specifically after TBI, found that interventions incorporating feedback and task-performance analysis were preferable for improving self-awareness. They caution, however, that the overall quality of evidence remains low and heterogeneous, precluding definitive clinical recommendations. The extent to which these conclusions generalise to the chronic phase or to other ABI aetiologies remains to be established.

The evidence reviewed above (63, 64) favours self-awareness training that directly engages feedback-driven self-monitoring over EF training pursued independently of it. One candidate explanation, following from the model’s own logic, concerns the demand-specific nature of adaptive coding. Because prefrontal neurons recode whatever information a task currently requires, EF training that does not itself demand self-monitoring would exercise the same general mechanism on other, non-self-relevant dimensions, with no guarantee of transfer to self-relevant representations. This is a theoretical account of why the evidence above might take this form, not additional support for it, and remains untested.

### 6.4 Future directions

Several questions raised by the integrated framework proposed here remain unexplored and merit direct empirical attention. First, the model’s central mechanistic claims, that adaptive coding and precision-weighted prediction error processing jointly underlie the EF-insight relationship, have not been directly tested in ABI populations. The neuroimaging evidence reviewed in Section 3 establishes that relevant prefrontal and salience-network regions are implicated in both EF and awareness, but does not yet provide direct evidence for the specific computational mechanisms (adaptive population coding, precision weighting) the integration proposes. Second, as noted in Section 6.3, whether EF-targeted rehabilitation produces measurable collateral gains in self-awareness, as the model predicts, has not been directly tested and would benefit from trials with pre-registered awareness outcome measures rather than EF outcomes alone. Third, longitudinal studies tracking EF and self-awareness recovery trajectories together, rather than cross-sectionally, would help establish whether the two constructs recover in the coupled manner the integrated account predicts, or dissociate over the recovery course. Finally, the present review has concentrated on executive and computational contributions to impaired self-awareness; integrating emotional, motivational, and social-cognitive contributions into this mechanistic account, rather than treating them as separate from it, represents an important extension beyond the scope of the current paper.

## 7 Conclusion

The relationship between executive functions and self-awareness in acquired brain injury is clinically significant and theoretically important. Across diverse populations, assessment approaches, and injury aetiologies, executive dysfunction is consistently associated with impaired self-awareness, with the strength of the relationship varying by EF domain, assessment method, injury severity, and time post-injury. The structured interview-based assessment of insight, capturing intellectual and emergent awareness across functional domains, shows the strongest and most consistent relationships with EF measures, and should be preferred over self-proxy discrepancy scores in clinical and research contexts.

Four theoretical frameworks—the Cognitive Awareness Model, the Dynamic Comprehensive Model of Awareness, predictive coding, and adaptive coding—each captures important aspects of this relationship. We have proposed that the adaptive coding and predictive coding frameworks are complementary and jointly offer a candidate mechanistic account, though its specific claims have not yet been directly tested in ABI populations. Predictive coding specifies the computational logic: self-awareness depends on precision-weighted prediction error processing that drives updating of hierarchical self-models. Adaptive coding specifies the neural mechanism: prefrontal neurons flexibly code self-relevant information when self-monitoring is contextually demanded, such that damage to prefrontal networks would be expected to disrupt this capacity and simultaneously impair executive functions and insight. This integration situates the EF-insight relationship in prefrontal systems, as a theoretical alternative to treating EF and insight as correlated but distinct capacities, and generates testable predictions about the direction and level at which disruption occurs following ABI.

The clinical and rehabilitation implications of this integrated account are substantive, though they vary in evidentiary support. Assessment should be multi-method, domain-specific, and attentive to the distinction between intellectual, emergent, and anticipatory awareness. Rehabilitation should be calibrated to the patient’s current awareness level, with metacognitive strategies reserved for patients with sufficient residual adaptive coding capacity to benefit from them, and self-awareness specific intervention favoured over EF training as the primary route to improving insight. Two further implications follow from the theoretical model rather than from direct evidence in ABI populations, and are offered as testable predictions rather than established recommendations. Training conducted across varied contexts may build adaptive coding capacity itself, rather than merely revealing context-dependent performance that was present all along; and feedback delivered within active task performance, and embedded in activities of motivational significance to the patient, may maximise its precision weighting and impact on self-model updating.
